# *ZmbHLH124* identified in maize recombinant inbred lines contributes to drought tolerance in crops

**DOI:** 10.1111/pbi.13637

**Published:** 2021-07-06

**Authors:** Shaowei Wei, Ran Xia, Chengxuan Chen, Xiaoling Shang, Fengyong Ge, Huimin Wei, Huabang Chen, Yaorong Wu, Qi Xie

**Affiliations:** ^1^ State Key Laboratory of Plant Genomics Institute of Genetics and Developmental Biology The Innovative Academy of Seed Design Chinese Academy of Sciences Beijing China; ^2^ University of Chinese Academy of Sciences Beijing China

**Keywords:** drought stress, maize, recombinant inbred lines, RNA‐seq, *ZmbHLH124*

## Abstract

Due to climate change, drought has become a severe abiotic stress that affects the global production of all crops. Elucidation of the complex physiological mechanisms underlying drought tolerance in crops will support the cultivation of new drought‐tolerant crop varieties. Here, two drought‐tolerant lines, RIL70 and RIL73, and two drought‐sensitive lines, RIL44 and RIL93, from recombinant inbred lines (RIL) generated from maize drought‐tolerant line PH4CV and drought‐sensitive line F9721, were selected for a comparative RNA‐seq study. Through transcriptome analyses, we found that gene expression differences existed between drought‐tolerant and ‐sensitive lines, but also differences between the drought‐tolerant lines, RIL70 and RIL73. *ZmbHLH124* in RIL73, named as *ZmbHLH124* ^T‐ORG^ which origins from PH4CV and encodes a bHLH type transcription factor, was specifically up‐regulated during drought stress. In addition, we identified a substitution in *ZmbHLH124* that produced an early stop codon in sensitive lines (*ZmbHLH124* ^S‐ORG^). Overexpression of *ZmbHLH124* ^T‐ORG^, but not *ZmbHLH124* ^S‐ORG^, in maize and rice enhanced plant drought tolerance and up‐regulated the expression of drought‐responsive genes. Moreover, we found that ZmbHLH124^T‐ORG^ could directly bind the *cis*‐acting elements in *ZmDREB2A* promoter to enhance its expression. Taken together, this work identified a valuable genetic locus and provided a new strategy for breeding drought‐tolerant crops.

## Introduction

Maize (*Zea mays* L.) is grown more than any other staple cereal with the current grain production at about 1 billion tons/year globally. The majority of maize germplasms are produced in rain‐fed regions and susceptible to drought, and maize is sensitive to drought and intolerant of water‐deficient soils because of its shallow roots. Thus, water scarcity can lead to huge reduction in maize yield, limit maize expansion outside present‐day agriculture areas, and trigger enormous social problems (Chaves and Oliveira, 2004; Fang and Xiong, 2015). Given ongoing climate crisis, the development of maize germplasm with enhanced drought tolerance and higher water use efficiency (WUE) has become an urgent goal for major breeding programs (Ribaut *et al*., 2009).

Previous studies in *Arabidopsis* and rice have elucidated multiple drought response pathways and metabolic networks. The processes include stress signal perception, signal transduction and amplification, and adjustments at the whole‐plant level. Genes encoding various transcription factor (TF) family members, such as ABA‐responsive element‐binding proteins/factors (AREBs/ABFs), dehydration‐responsive element‐binding factors (DREBs), basic leucine zipper (bZIP) proteins, zinc‐finger proteins, and NAC (NAM‐ATAF‐CUC2) TFs, play important roles as hubs of transcription networks and have been used to elicit multiple biochemical and developmental pathways to improve plant performance during drought stress (Kuromori, 2014; Hu and Xiong, 2014; Seki *et al*., 2007; Zhu, 2016). To date, a few studies have been conducted to reveal how maize responds to drought stress and several new key components have been discovered to modulate plant response to drought stress (Kakumanu *et al*., 2012; Liu *et al*., 2015; Thatcher *et al*., 2016; Zhang *et al*., 2014). Basic helix‐loop‐helix (bHLH) proteins are found in the three eukaryotic kingdoms (fungi, plants and animals) and constitute the second largest family of TFs in plants, but their function in drought tolerance remains poorly described (Carretero‐Paulet *et al*., 2010; Feller *et al*., 2011). Three bHLH TFs, ZmPIF3 (Gao *et al*., 2015), ZmPIF1 (Gao *et al*., 2018), and ZmPTF1 (Li *et al*., 2019), have been reported to be involved in drought response in maize. Currently, identifying the genetic components underlying drought tolerance is still considered to be challenging in maize owing to the complexity of genome (Jiao *et al*., 2017; Schnable *et al*., 2009) and the multi‐strategic nature of drought response. However, RNA‐seq is a powerful method for understanding transcriptomic dynamics across different tissues or conditions with little background noise, concise normalization of data sets, and relatively low cost (Hrdlickova *et al*., 2017; Wang *et al*., 2009).

In this study, we screened four lines with different drought‐responsive phenotypes isolated from a recombinant line population for RNA‐seq analysis. RIL44 and RIL93 were drought‐sensitive lines, whereas RIL70 and RIL73 were drought‐tolerant lines. After comparative transcriptomic analyses, we found that the drought tolerance mechanism of RIL73 was different from that of RIL70. According to gene expression patterns, we identified a bHLH type TF, named ZmbHLH124, involved in drought response of maize. Transgenic analysis showed that *ZmbHLH124* ^T‐ORG^ overexpression in both rice and maize improved survival rates and decreased water loss rates when plants were treated with drought treatment. Moreover, ZmbHLH124^T‐ORG^ could bind recognition sites in the promoter of *ZmDREB2A,* a well‐known drought response TF, and promote its expression to enhance drought tolerance in plants. Our study provided a new avenue to discover drought‐responsive genes and identified a valuable signalling protein for maize breeding.

## Results

### Phenotypic and physiological characteristics of RILs responding to drought stress

**Figure 1:**
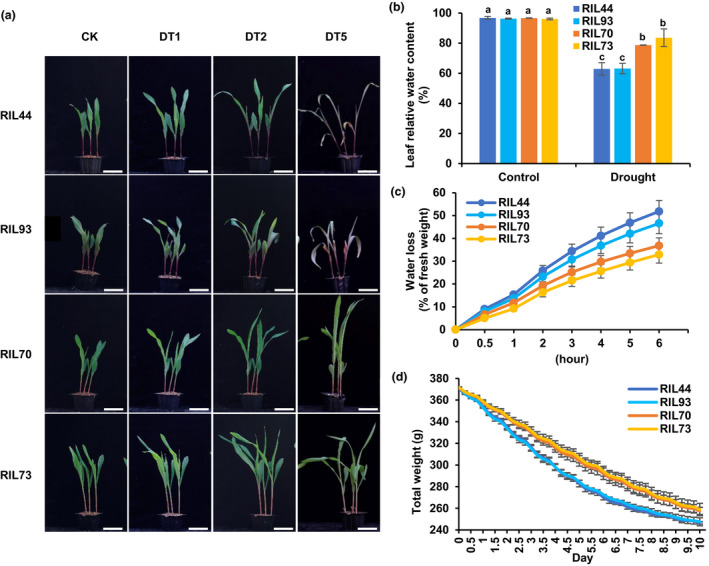
The physiological characteristics of four different lines in response to drought stress. (a) Phenotypic changes of four lines during drought treatment. CK, DT1, DT2, and DT5 refer to drought treatment for 0, 1, 2 and 5 days, respectively. Bar = 5 cm. (b) Leaf relative water content. The leaves were collected when the soil moisture content was about 86% (control) and 12% (drought). Statistical significance was determined by one‐way ANOVA Duncan’s multiple range tests (*P* < 0.05). (c) Water loss rates of detached leaves. (d) Water loss rates of the whole seedlings during drought treatment. Y‐axis represents the total weight of pots, soil and seedlings. Error bars, s.d., calculated from the results of at least three independent experiments.

The contribution of natural variation to stress tolerance in plants has been observed in many plant species. To discover genetic components related to drought tolerance in maize, a population of recombinant inbred lines (RILs), together with their parental lines, the drought‐tolerant line PH4CV and the drought‐sensitive line F9721 (Figure S1), were grown to perform drought tolerance screening at the seedling stage (Min *et al*., 2016). Four lines with contrasting drought response phenotypes, two sensitive lines and two tolerant lines, were selected for physiological and molecular analysis to try to discover the genes involved. After one day without water (DT1), there were no obvious phenotype differences among the four lines, while after 2 days (DT2), sensitive lines exhibited growth inhibition compared to tolerant lines. This difference was particularly distinct after 5 days (DT5) when sensitive lines showed wilted and curled leaves while leaves of tolerant lines remained green and erect. As shown in Figure 1a, RIL44 and RIL93 are drought‐sensitive lines while RIL70 and RIL73 are drought‐tolerant lines.

Leaf water content is an important parameter used for determining plant drought tolerance, and guiding fertilizer application and irrigation regimens (Jin *et al*., 2017). Plants tolerant to drought reliably show stable water content, therefore, we measured the water retention capacity of the four lines. The leaf relative water contents (LRWC) of tolerant lines were higher than those of sensitive lines when they were grown in drought conditions with a soil moisture level of 12%, while the LRWC of four lines showed no obvious differences under well‐watered conditions with a soil moisture level of 86% (Figure 1b). The detached leaves of tolerant lines, RIL70 and RIL73, lost water slower than those of the sensitive lines, RIL44 and RIL93 (Figure 1c). To estimate water transpiration during drought treatment, we further examined the weight loss of plants grown in soil. Figure 1d showed a continuous water loss pattern of seedlings during a 12‐day period. Starting from equal weight and without additional water supplementation, RIL70 and RIL73 lost less weight than RIL44 and RIL93. These results indicated that RIL70 and RIL73 could maintain a more stable water status than RIL44 and RIL93 under water deficit.

### Differential analysis of chromosome regions constructed by bin map through RNA‐seq

The different drought response phenotypes of the four RILs could be due to the rearrangement and combination of parental genomes. To better understand RIL genetic diversity and transcriptome changes during drought treatment, RNA‐seq was performed to facilitate the comparative analysis. Leaves of the four RILs sampling at four‐time points (CK, DT1, DT2, and DT5) during drought treatment were harvested and the total RNAs were extracted for RNA‐seq. Two biological replicates per RIL at each time point were used for sequencing and reducing experimental errors. After measuring the gene expression level correlation among replicates, the Pearson correlation coefficient’s square was at least above 0.96 (*R* ^2^ > 0.96) between two replicates of each sample (Figure S2), indicating that expression patterns of each sample between two replicates were significantly similar and the data was suitable and reliable for subsequent studies.

First, SNPs of each RIL were identified from RNA‐seq data by using maize B73 RefGen_v3 as the reference genome. Then, the four RIL genotypes (Table S1) were called according to the physical positions of SNPs. Because of the lack of detailed parental genome information, we could not specify the genetic sources of individual SNPs, that is to say, at least two genotypes were needed to distinguish genetic backgrounds and find regions involved in stress response. Thus, we combined four genotypes into one file for comparative analysis. In order to find the differences in response to drought stress among RILs, or the uniqueness of drought response of each RIL, we constructed bin maps (Figure S3) of RIL44, RIL93, RIL70, and RIL73 without parental genotypes by using a modified sliding window method (Huang *et al*., 2009) to discover each RIL’s specific chromosome regions after excluding the common regions shared by any of the other three RILs in our study. Bin maps could be homozygous or heterozygous depending on the types of SNPs in the sliding windows. Finally, 15 regions of the four types were detected in total by comparing these bin maps (Table 1), including the specific region of each RIL, common regions between two RILs, or among three RILs, and common regions of all four RILs.

**Table 1:** Gene numbers in different chromosome regions among four RILs

Region no.	Region type	DEG number in these regions	Total gene number in these regions
1	RIL44 specific regions different from other three RILs	162	7057
2	RIL93 specific regions different from other three RILs	684	5161
3	RIL70 specific regions different from other three RILs	89	6540
4	RIL73 specific regions different from other three RILs	488	8923
5	common regions between RIL44 and RIL93 different from RIL70 or RIL73 background	903	6951
6	common regions between RIL44 and RIL70 different from RIL73 or RIL93 background	168	5946
7	common regions between RIL44 and RIL73 different from RIL70 or RIL93 background	973	14 492
8	common regions between RIL93 and RIL70 different from RIL73 or RIL93 background	2286	18 476
9	common regions between RIL70 and RIL73 different from RIL44 or RIL93 background	381	6065
10	common regions between RIL73 and RIL93 different from RIL44 or RIL70 background	1292	8933
11	common regions among RIL70, RIL44 and RIL93 different from RIL73 background	662	4852
12	common regions among RIL70, RIL44 and RIL73 different from RIL93 background	313	3909
13	common regions among RIL70, RIL73 and RIL93 different from RIL44 background	548	3806
14	common regions among RIL44, RIL73 and RIL93 different from RIL70 background	528	3432
15	common regions among four RILs	42	188

DEG, Differentially Expressed Gene.

### Analysis of differentially expressed TFs in RIL73 specific regions

To discover genes related to drought tolerance in maize, we followed with interest in specific regions in the drought‐tolerant lines RIL70 and RIL73. We found that RIL73 had a higher capacity for leaf water retention than RIL70 (Figure 1b,c); therefore, we speculated that there could be regions specific in RIL73 for this phenotype. Therefore, genes in specific regions of RIL73 (red boxes in Figure S3) were targets for further analysis.

**Figure 2:**
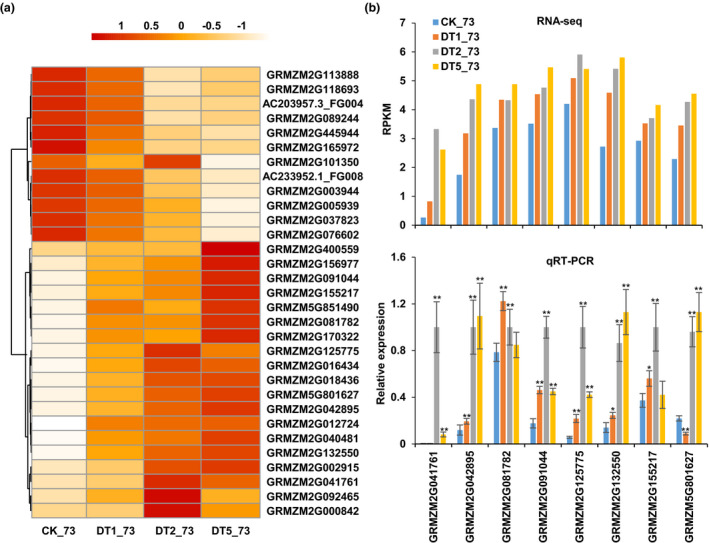
Differentially expressed transcription factors in specific regions from RIL73. (a) Heat map of transcription factors with differential expression in specific regions. Colour was determined by Z score. (b) Validation of RNA‐seq results by qRT‐PCR. The top half is the result of RNA‐seq, the bottom half is the result of qRT‐PCR. RPKM: Reads Per Kilobase per Million mapped reads. Error bars, s.d., calculated from the results of at least three independent experiments. Statistical significance compared with CK was determined by a two‐sided *t*‐test: *P < 0.05, **P < 0.01.

Among the 488 differentially‐expressed genes (DEGs) in specific regions of RIL73 (Table 1), 31 DEGs were found to have DNA binding activity and/or TF activity based on gene annotations (Table S2). According to gene expression patterns, they were divided into two clusters: one cluster was repressed genes and the other was induced genes under drought stress (Figure 2a). After searching the detailed information of homologous genes of these 31 TFs, eight genes with exhibiting upregulation by drought treatment, *GRMZM2G041761*, *GRMZM2G042895*, *GRMZM2G081782*, *GRMZM2G091044*, *GRMZM2G125775*, *GRMZM2G132550*, *GRMZM2G155217*, and *GRMZM5G801627* annotated as stress response, drought tolerance, or unknown, were selected to validate the accuracy and reproducibility of RNA‐seq data by quantitative real‐time PCR (qRT‐PCR). The result showed that the expression of these genes was up‐regulated by drought stress (Figure 2b). In addition, among the four unknown function genes, *GRMZM2G041761*, *GRMZM2G042895*, *GRMZM2G091044*, and *GRMZM2G132550*, only *GRMZM2G132550* from RIL73 had the same genetic background as PH4CV, and the genetic backgrounds of the other three genes were the same as that of drought‐sensitive line F9721. Thus, we chose *GRMZM2G132550* as the candidate gene for drought analysis.

### *ZmbHLH124* is involved in drought tolerance in plants

**Figure 3:**
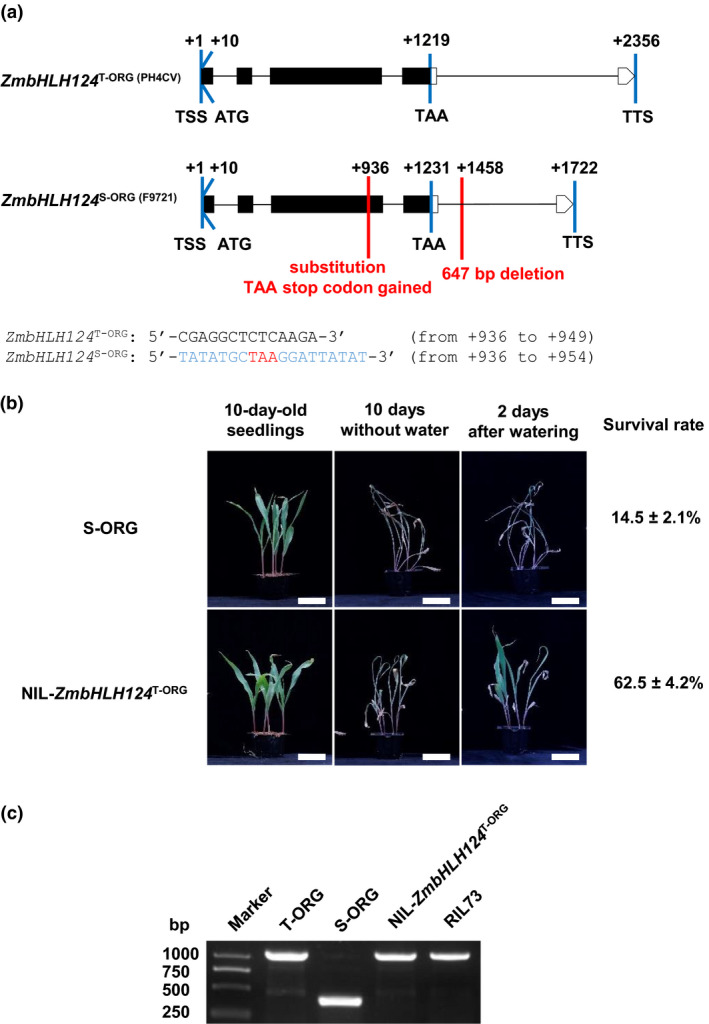
*ZmbHLH124* is involved in drought response in maize seedlings. (a) Schematic diagram of *ZmbHLH124* transcript regions. Black boxes represent exons, straight lines represent introns and white frames represent 5′ or 3′UTR. The highlight red lines and annotations are main differences which distinguish *ZmbHLH124* ^S‐ORG^ from *ZmbHLH124* ^T‐ORG^. The Indel +936 that causes premature termination of translation are labelled. TSS: transcript start site; TTS: transcript terminal site. (b) Comparison of drought response of parental line S‐ORG and NIL‐*ZmbHLH124* ^T‐ORG^. Survival rates were calculated from the results of at least three independent experiments, *n* ≥ 12. Bar = 5 cm. (c) The molecular marker (647 bp deletion) selection of segregating NILs carrying a homozygous allele of *ZmbHLH124* ^T‐ORG^.

*GRMZM2G132550* encodes a basic helix‐loop‐helix (bHLH) type TF referred to as *ZmbHLH124* in MaizeGDB (https://www.maizegdb.org/). Thus, we designated this gene as such and will heretofore refer to it in this fashion. We amplified and sequenced the genomic sequence of *ZmbHLH124* from two parental lines, *ZmbHLH124* ^T‐ORG^ originally from the drought‐tolerant line PH4CV (T‐ORG) and *ZmbHLH124* ^S‐ORG^ from the drought‐sensitive line F9721 (S‐ORG). In comparison to *ZmbHLH124* ^T‐ORG^, two main variants, indel +936 (5 bp) and indel +1458 (647 bp), were discovered downstream from the transcript start site in *ZmbHLH124* ^S‐ORG^ (Figure 3a). Indel +936 was a short fragment substitution, in *ZmbHLH124* ^T‐ORG^, the nucleotide sequences from +936 to +949 were 5′‐CGAGGCTCTCAAGA‐3′, while in the *ZmbHLH124* ^S‐ORG^, sequences from +936 to +949 were 5′‐TATATGCTAAGGATTATAT‐3′, which resulted in an immediate TAA translation stop codon gained in *ZmbHLH124* ^S‐ORG^. Indel +1458 was a 647 bp deletion in the fourth intron of *ZmbHLH124* ^S‐ORG^, and adapted as a marker to screen near‐isogenic lines (NIL). From the structural changes caused by DNA substitution, we speculated that *ZmbHLH124* ^S‐ORG^ might lose its function because of the premature stop codon which might disrupt the dimerization domain of the predicted bHLH TF (Figure S4). To verify whether *ZmbHLH124* ^T‐ORG^ can complement the lost function of *ZmbHLH124* ^S‐ORG^ in maize line S‐ORG, we introgressed the *ZmbHLH124* ^T‐ORG^ into the S‐ORG by generating NIL‐*ZmbHLH124* ^T‐ORG^ through five generations of successive backcrossing of the F_1_ plants (RIL73 × S‐ORG). For each generation, the *ZmbHLH124*‐heterozygous plants were selected and backcrossed with S‐ORG. After two generations of self‐pollination, we obtained BC_5_F_3_ plants carrying the homozygous‐tolerant *ZmbHLH124* ^T‐ORG^ allele (Figure S5). Evaluations of drought tolerance confirmed that the NIL‐*ZmbHLH124* ^T‐ORG^ plants were more tolerant than S‐ORG seedlings (Figure 3b,c). These results indicated that the intact *ZmbHLH124* ^T‐ORG^ contributed to drought tolerance in maize germplasm T‐ORG.

**Figure 4:**
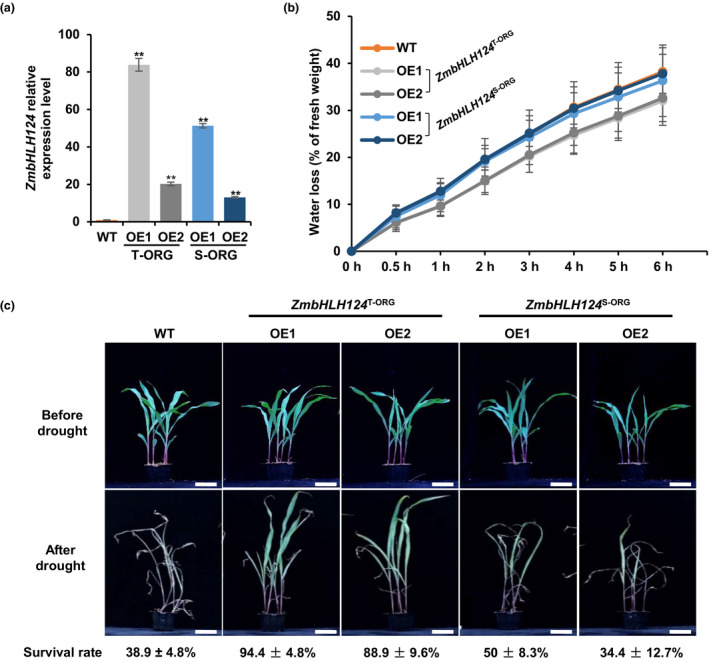
Phenotypes of *ZmbHLH124* transgenic maize upon drought treatment. (a) Relative expression levels of *ZmbHLH124* detected by qRT‐PCR in maize grown under normal conditions. The data were based on three independent biological replicates. Error bars, s.d.. Statistical significance compared with WT was determined by a two‐sided *t*‐test: *P < 0.05, **P < 0.01. (b) Water loss rates of detached leaves. Error bars, s.d., calculated from the results of at least three independent experiments. (c) Drought treatment of transgenic maize as compared to WT in different time points. Survival rates were calculated from the results of at least three independent experiments, *n* ≥ 12. Bar = 5 cm.

Given that drought stress up‐regulated *ZmbHLH124* expression, we generated both transgenic rice and maize, overexpressing the cDNAs of *ZmbHLH124* from both T‐ORG and S‐ORG lines to verify whether *ZmbHLH124* ^T‐ORG^ could generally improve monocot drought tolerance. For the transgenic maize, the transcript levels of the transgenes were verified by qRT‐PCR (Figure 4a). We confirmed the full‐length mRNA and protein using semi‐qPCR and western blot assays (Figure S6). Then, two independent transgenic overexpression lines of each genotype were selected for further analysis. The detached leaves of *ZmbHLH124* ^T‐ORG^ transgenic plants lost water much slower than those of transgene‐free sibling plants (WT) or *ZmbHLH124* ^S‐ORG^ overexpression (OE) plants (Figure 4b). But no significant differences were found in stomatal density and aperture among the transgenic lines and the WT (Figure S7). Upon drought treatment, more than 85% of *ZmbHLH124* ^T‐ORG^ transgenic maize plants survived; whereas, the survival rates of WT and *ZmbHLH124* ^S‐ORG^ transgenic plants were approximately 38.9% and below 50%, while no notable changes were observed in the transgenic maize compared to WT under normal growth conditions (Figure 4c), indicating that *ZmbHLH124* ^T‐ORG^ OE improved drought tolerance in maize.

Similarly, improved drought tolerance was also observed in transgenic rice transformed with *ZmbHLH124* driven by the *ZmUbiquitin1* promoter. The transcript levels of transgenes were verified by qRT‐PCR (Figure S8a), and the phenotypes of two independent *ZmbHLH124* transgenic lines with detected high transgene expression per parental source were determined. In comparison to WT and *ZmbHLH124* ^S‐ORG^ OE plants, the *ZmbHLH124* ^T‐ORG^ OE rice displayed significantly enhanced drought tolerance, while remarkable morphological changes were not observed under standard growth conditions for all transgenic lines. The survival rates of WT plants and *ZmbHLH124* ^S‐ORG^ OE plants were around 11% and below 20%, respectively, more than 45% of *ZmbHLH124* ^T‐ORG^ OE plants were alive in the parallel water‐withholding experiments (Figure S8b), suggesting that *ZmbHLH124* ^T‐ORG^ OE could improve rice drought tolerance. Collectively, these data suggested that the single intact gene *ZmbHLH124* ^T‐ORG^ could improve drought tolerance in monocot plants including rice and maize.

### ZmbHLH124 activates *ZmDREB2A* expression by directly binding to its promoter

**Figure 5:**
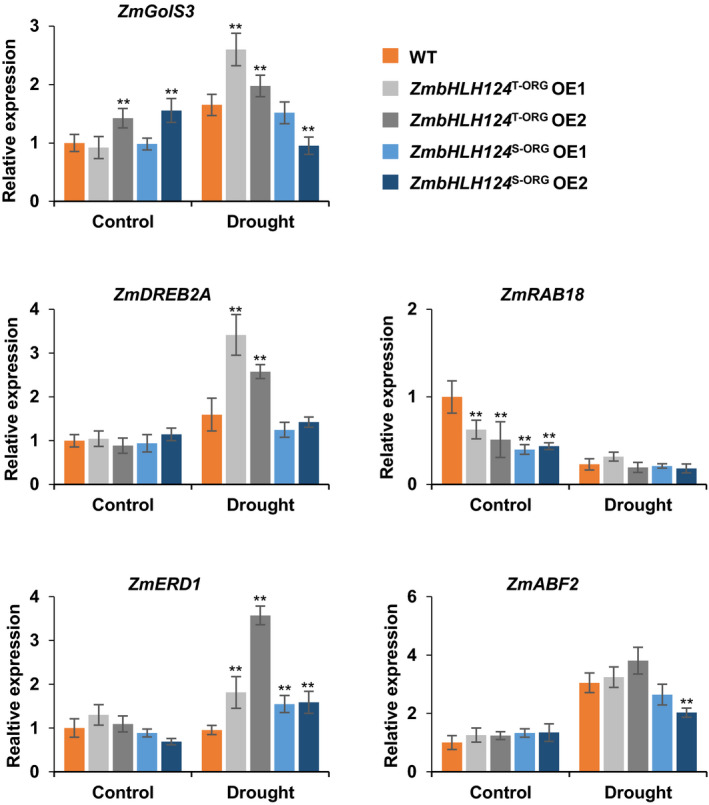
Relative expression levels of candidate drought‐inducible genes detected by qRT‐PCR in maize under control and drought conditions. The data were based on three independent biological replicates. Error bars are s.d.. Statistical significance compared with WT was determined by a two‐sided *t*‐test: *P < 0.05, **P < 0.01.

*ZmbHLH124* encodes a bHLH type TF and it is known that such a TF can bind bHLH recognition core sequences (5′‐CANNTG‐3′) named E‐box in the promoter of downstream genes to activate or repress their transcriptions (Nakata *et al*., 2013; Song *et al*., 2013; Tian *et al*., 2019). Thus, some downstream genes should be regulated in response to the expression of *ZmbHLH124*. In order to discover the target genes, transcript levels of several drought‐inducible candidate genes of maize homologs, such as *DREB2A*, *ERD1*, *GolS3*, *RAB18*, and *ABF2* (Mao *et al*., 2015; Zhu *et al*., 2020) containing copies of bHLH recognition core sequence in their promoters, were detected in the transgenic maize lines with or without exposure to drought treatment. Our qRT‐PCR results showed that two days after drought treatment, *ZmRAB18* was not induced by drought stress and the transcript levels of *ZmDREB2A*, *ZmERD1*, and *ZmGolS3* in both *ZmbHLH124* ^T‐ORG^ transgenic maize lines were induced to higher levels than those of WT or *ZmbHLH124* ^S‐ORG^ transgenic maize lines. It had been reported that *ERD1* induction required the concerted action of ZFHD1 and NAC TFs (Tran *et al*., 2007), and *GolS3*‐functioned downstream of *DREB2A* (Qin *et al*., 2007; Sakuma *et al*., 2006a). *ZmABF2* was also up‐regulated in maize upon drought treatment, but its transcript levels in *ZmbHLH124* ^T‐ORG^ transgenic maize lines were not significantly higher than those of WT or *ZmbHLH124* ^S‐ORG^ transgenic maize lines (Figure 5).

**Figure 6:**
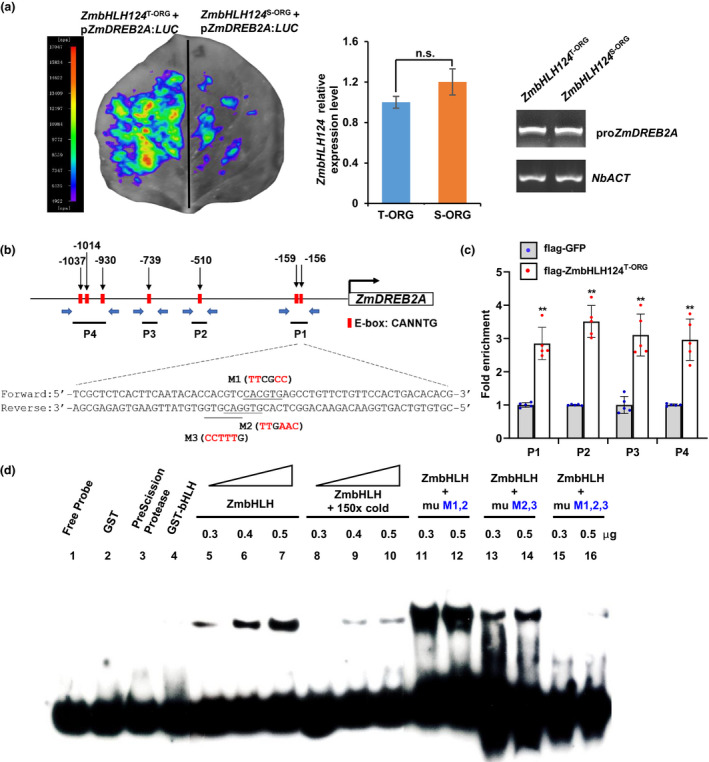
ZmbHLH124 directly activates expression of *ZmDREB2A*. (a) Transactivation assay in tobacco leaf by transient expression. Left: firefly luciferase activity detection; middle: the relative expression levels of *ZmbHLH124*; right: PCR‐DNA amount of promoters to represent the equal plasmid DNA in agro‐infiltration were applied between parallel experiments, and *NbACT* was employed as the internal control in the DNA analysis. The data were based on three independent biological replicates. (b) Schematic diagram of the *ZmDREB2A* promoter. Red boxes indicate the putative E‐box motifs. (c) ChIP‐qPCR identified significant enrichment in the fragment containing the E‐box motif in the *ZmDREB2A* promoter. The fragments used in ChIP‐qPCR are indicated in (b). Fold enrichments were calculated relative to input. (d) ZmbHLH124^T‐ORG^ directly bound to the promoter of *ZmDREB2A in vitro*. Lane 1–4: control; lane 5–10: protein and DNA interaction; lane 11–16: protein and mutated DNA interaction. Mutations of binding sites are labelled in (b). mu: mutation. Error bars, s.d., calculated from the results of at least three independent experiments. Statistical significance was determined by a two‐tailed *t*‐test: **P < 0.01; n.s.: not significant.

A previous study demonstrated that ZmDREB2A functioned as a transcriptional activator and played an important role in plant stress tolerance and that overexpression of *ZmDREB2A* resulted in improved drought tolerance in *Arabidopsis* (Qin *et al*., 2007). To further confirm whether *ZmbHLH124* could up‐regulate the expression of *ZmDREB2A in vivo*, a transient expression system was employed to investigate its transcription activation effect (Shu *et al*., 2013). We constructed the luciferase (LUC) reporter plasmids driven by *ZmDREB2A* promoter, p*ZmDREB2A*:*LUC*, and the effector plasmids *ZmbHLH124* ^T‐ORG^ and *ZmbHLH124* ^S‐ORG^ driven by the CaMV *35S* promoter, respectively. When the p*ZmDREB2A*:*LUC* construct combined with *ZmbHLH124* ^T‐ORG^ was co‐transformed in *Nicotiana benthamiana*, strong LUC activity was detected. However, when p*ZmDREB2A*:*LUC* was combined with an equal amount of effector *ZmbHLH124* ^S‐ORG^, the LUC activity was particularly weak (Figure 6a). This result indicated that *ZmbHLH124* ^T‐ORG^ indeed promoted *ZmDREB2A* expression *in vivo*.

To check whether *ZmbHLH124* ^T‐ORG^ directly binds to the *ZmDREB2A* promoter *in vivo* and *in vitro*, chromatin immunoprecipitation (ChIP)‐qPCR assay and electrophoretic mobility shift assay (EMSA) were performed to determine the interaction between *ZmbHLH124* ^T‐ORG^ and the *ZmDREB2A* promoter. First, we identified seven putative E‐box motifs within 1.2 kb upstream of the *ZmDREB2A* transcription start site, then four fragments (P1–4) were selected to be amplified by qPCR using specific primer pairs that spanned the E‐box motifs (Figure 6b). The ChIP‐qPCR assays showed that flag‐ZmbHLH124^T‐ORG^ had a 2‐to‐4‐fold enrichment of P1‐4 regions compared with flag‐GFP (Figure 6c), suggesting that *ZmDREB2A* was a direct target of ZmbHLH124^T‐ORG^ *in vivo*. Next, based on the ChIP‐qPCR results, EMSAs were employed to confirm further the interaction between ZmbHLH124^T‐ORG^ and *ZmDREB2A* promoter *in vitro*. The recombinant GST‐ZmbHLH124^T‐ORG^ fusion protein was expressed and purified from *E*. *coli*, one fragment of P1 region was chosen as a probe for this assay. In addition, the fusion protein was digested by a protease to release the bHLH protein from the GST tag before performing EMSA assays (Figure 6d, lane 2–4). The results revealed that the mobility of the probe was significantly delayed in the presence of ZmbHLH124^T‐ORG^ (Figure 6d, lane 1, 5–7). Further, the cold‐competitor probes (excess of unlabelled fragments) were sufficient to compete for the ZmbHLH124^T‐ORG^ binding activity (Figure 6d, lane 8–10). Moreover, in addition to the two E‐box motifs, there is another binding motif called non‐E‐box motif (5′‐NACGTG‐3′) (Li *et al*., 2006; Toledo‐Ortiz *et al*., 2003) in the probe (Figure 6b), so we had mutated three bHLH binding sites in different combinations to test TF binding. Only when all motifs were mutated did the binding activity of ZmbHLH124^T‐ORG^ disappear (Figure 6d, lane 11–16). The EMSA results demonstrated that ZmbHLH124^T‐ORG^ directly bound to the *ZmDREB2A* promoter. Taken together, these data suggested the *ZmbHLH124* ^T‐ORG^ could directly bind to the *ZmDREB2A* promoter to promote its expression to improve plant drought tolerance.

## Discussion

Drought resistance is a complex trait due to the simultaneous occurrence of other stresses and the multiple stress response strategies in plants (Hu and Xiong, 2014; Takeda and Matsuoka, 2008). In past decades, hundreds of genes in different pathways had been identified with the purpose of understanding how plants adapt to drought stress, thus providing genetic materials and targets for crop improvement. However, the detailed molecular and biochemical mechanisms underlying drought tolerance remain largely unclear (Basu *et al*., 2016; Deikman *et al*., 2012; Fang and Xiong, 2015). In the present study, four individuals from a panel of RILs, drought‐sensitive lines RIL44 and RIL93 and drought‐tolerant lines RIL70 and RIL73, were used based on the survival rates of maize seedlings upon drought treatment. RIL70 and RIL73 had higher water use efficiency (WUE) than RIL44 and RIL93 (Figure 1), and those lines could be used for identification of drought response‐related genes. Genetic components with respect to drought response within parental lines must have been rearranged among segregated RIL individuals. Due to loci homozygosity established by repeated self‐pollination, RILs can serve as powerful tools for genetic analysis and for scoring invasive phenotypes in a variety of situations (Broman, 2005; Candela and Hake, 2008). Additionally, factors such as overdominance, epistasis, and hybrid vigour contribute to transgression in RILs but are not influential as in F_2_ populations (deVicente and Tanksley, 1993). Therefore, it is relatively easy and straightforward to study drought tolerance in maize.

The strategy for identifying novel genetic loci determining maize drought tolerance described here is based on the subtraction of similar regions shared by any of the remaining three RILs from RIL73 by means of bin map construction to locate specific regions of RIL73 that might confer drought tolerance. This is an exploratory study using available materials and data to mine valuable genetic components. Here we use the SNPs identified from transcriptome data to construct bin maps, mainly taking into account the maize genome size and complexity. Bin construction can connect scattered SNPs into fragments for distinguishing the chromosomal composition of different RILs in a parent‐independent way. Such treatment can not only reduce the errors caused by sequencing, but also increase the accuracy of subtractive analysis. Moreover, subtractive analysis is applied to locate specific regions in transgressive individual, like RIL73, in which alleles that are involved in drought tolerance with similar or additive effects are very likely to converge. In addition, based on our observation that RIL73 had stronger water retention capacity than RIL70, the DEGs from specific regions (No.4 in Table 1 and red boxes in Figure S3) of RIL73 are likely unique and worth further analysis in our study. Our work provides a useful trait research method for the study of species with complex and large genome, and even without reference genome information.

*ZmbHLH124* was discovered from a specific region of RIL73, and its expression continued to be up‐regulated during drought stress (Figure 2). *ZmbHLH124* ^T‐ORG^ not only complemented the function of *ZmbHLH124* ^S‐ORG^ relevant to drought tolerance by introgression into the S‐ORG background but also improved drought tolerance through its overexpression in maize and rice. Therefore, despite of its similar function between different crops, *ZmbHLH124* ^T‐ORG^ must play a key role in the drought response signalling pathway by regulating known and conserved target genes involved in drought response. The bHLH type TFs target promoters of downstream genes, including other TFs. DREB2A is an important and versatile regulator in stress response, a few studies have reported that its homologs can mediate the expression of genes which respond to certain abiotic stresses in plants, with the role of DREB2A in drought response being most studied (Mallikarjuna *et al*., 2011; Mizoi *et al*., 2013; Qin *et al*., 2007; Sakuma *et al*., 2006a; Sakuma *et al*., 2006b; Zhang *et al*., 2013). Similar to other plant species, *ZmDREB2A* may be a candidate gene targeted by *ZmbHLH124*. Based on tobacco leaf transient expression assays, ChIP‐qPCR and EMSA assays, ZmbHLH124^T‐ORG^ directly binds to the recognition sites in the promoter of *ZmDREB2A* to activate its expression, then ZmDREB2A regulates expression of water stress‐inducible genes to protect maize from drought stress, suggesting that *ZmbHLH124* acts upstream of *ZmDREB2A* in the osmotic‐stress signalling pathway. The downstream genes induced by DREB2A have been revealed to be a set of genes that encode late embryogenesis abundant proteins, which can reduce water loss rate as hydration buffers during stress, stabilize membrane structures, protect proteins and organelles, and sequestrate metal ions and reactive oxygen species (Banerjee and Roychoudhury, 2016; Battaglia *et al*., 2008; Hand *et al*., 2011; Yu *et al*., 2018). Perhaps that’s why *ZmbHLH124* has little to do with stomatal movement. Given that DREB2A is finely tuned at the transcriptional, posttranscriptional, and posttranslational level (Guerra *et al*., 2015; Kim *et al*., 2011; Morimoto *et al*., 2013; Morimoto *et al*., 2017; Qin *et al*., 2008), what we are concerned with is whether or how ZmbHLH124 interacts or cooperates with transcriptional regulators such as maize histone acetyltransferases, histone deacetylases (Zhao *et al*., 2014), and the GRF7 (Kim *et al*., 2012) homolog of maize to regulate the expression of *ZmDREB2A* tightly under normal and stress conditions.

In conclusion, through transgressive segregation in drought tolerance, four lines with different degrees of drought tolerance were identified from a maize RIL population and were used to perform RNA‐seq. Through SNP identification and comparative transcriptomic analyses of drought‐sensitive and ‐tolerant lines, we identified a specific gene locus that encodes a bHLH type TF, ZmbHLH124, which can activate the transcription of the drought‐related *ZmDREB2A* gene to contribute to drought tolerance in drought‐tolerant maize. Whether *ZmbHLH124* can improve drought tolerance during reproductive stages, which is very important for final crop yield and grain production, needs further study.

## Experimental procedures

### Plant materials and growth conditions

The maize (*Zea mays* L.) lines PH4CV, F9721, RIL44, RIL93, RIL70, RIL73, and C01 (wild type from China National Seed Group Co Ltd. used for transgenic lines construction) were used in this study. Maize seeds were directly planted in the soil and germinated in a temperature‐controlled greenhouse with natural light (day, 16 h, 26–28 °C; night, 8 h, 18–20 °C). Rice (*Oryza sativa* L.) ecotype Nipponbare was used for this study. After being soaked with 3% H_2_O_2_ at 28 °C for 24 h, the rice seeds were transferred to the water and germinated in a growth chamber (28 °C with dark condition), then were moved to the greenhouse.

### Water loss assays

For the drought treatment, 10‐day‐old maize seedlings and 4‐week‐old rice seedlings grown in soil under normal conditions in a greenhouse were used. After water had been withheld for 10–14 days, the phenotypic responses to drought stress were recorded. Three independent experiments were performed, with more than three replicate pots for each line in each experiment.

For the LRWC assay, the detached leaves were collected from maize plants grown in soil under normal conditions for ˜1 month, or under drought conditions when the moisture content was ˜12%. The detached leaves of maize plants were weighed (fresh weight, FW) immediately, then were placed in distilled water for 8 h or overnight and weighed again (saturated weight, SW). The water‐saturated leaves were dried to a constant weight (dry weight, DW) in a drying oven (105 °C for 30 min, then 70 °C for 12 h). Three biological replicates were assessed for each line. RWC = (FW − DW)/(SW − DW) × 100%.

For the water loss rate assay of detached leaves, the leaves of maize plants grown under normal conditions for 10 days were detached and immediately weighed, then placed in a culture chamber (26 °C, 50–55% relative humidity) and weighed at designated time intervals. Three biological replicates were assessed for each line. The percentage of loss of fresh weight was calculated on the basis of the initial weight of the leaves.

For the water transpiration assay, the maize plants had been grown in a culture chamber with conditions of 26 °C, 50–55% relative humidity for 2 weeks before measurement. Water loss of maize plants grown in soil was measured by weighing each pot with an electronic balance every 30 min for 15 days; the data were automatically recorded by a software that was connected to the balance (DroughtSpotter, Phenospex, Heerlen, The Netherlands). The experiments were repeated three times.

### RNA extraction, sequencing, and DEG analysis

Total RNA was isolated from the leaf tissues at different periods by a Total Plant RNA Extraction Kit (Karroten, Beijing, China). Sequencing libraries were generated using the NEBNext UltraTM RNA Library Prep Kit for Illumina (NEB). Poly(A)‐selected mRNA was sequenced on an Illumina HiSeq 2000 platform. RNA‐seq clean reads were aligned to the B73 version 3.0 reference genome by TopHat version 2.0.9 (Trapnell *et al*., 2009) allowing two segment mismatches. HTSeq version 0.6.1 (Anders *et al*., 2015) was used to count the read number of genes and the Reads Per‐Kilobase of the exon model per Million mapped reads (RPKM) was calculated based on the reads count and length of the respective gene. Differential expression analysis was performed by the DESeq R package (Anders, 2010). Genes with fold change ≥ 2 and a false discovery rate (FDR) adjusted *P* < 0.05 were selected as DEGs. Gene ontology (GO) analysis was performed by agriGO version 2.0 (Tian *et al*., 2017).

### SNP calling and line specific region comparison

RNA‐seq clean reads of each sample were aligned to the reference genome by STAR version 2.6.0 (Dobin *et al*., 2013). Bam files from the same lines at different periods were merged and underwent SNP calling via the Genome Analysis Toolkit (GATK, version 4.1.3) (McKenna *et al*., 2010). The SNPs with minimum depth ≥ 5 were selected for further analysis. The modified sliding window approach for bin map construction (Huang *et al*., 2009) was used to identify the line‐specific regions among RILs. Contiguous SNPs within 1 kb were classified into a window. The ratio of specific SNPs inside a window was calculated. The windows with more than 65% specific SNPs were regarded as specific windows. Contiguous specific windows were combined into a specific region.

### Quantitative real‐time PCR assays

Total RNA was isolated from the leaf tissues at different periods by an Ultrapure RNA Kit with DNaseI (CWBIO, Beijing, China) and was subjected to reverse transcription using a FastKing RT kit with gDNase (TIANGEN, Beijing, China). qRT‐PCR was performed using 2 × Talent qPCR PreMix (TIANGEN, Beijing, China) and the CFX96 real‐time system (Bio‐Rad, California, USA). Transcript levels were quantified at the logarithmic phase based on the expression of the housekeeping genes *ZmEF1A* (Lin *et al*., 2014) and *OsACTIN* (Shang *et al*., 2016) as internal controls. Three biological replicates were performed per line. Primer sequences for qRT‐PCR are shown in Table S3.

### Transformation vectors and generation of transgenic plants

Transgenic plants carrying constitutively expressing *ZmbHLH124* ^T‐ORG^ and *ZmbHLH124* ^S‐ORG^ were generated. To obtain the *Ubi‐ZmbHLH124* overexpression vector, an 888 bp *ZmbHLH124* ^T‐ORG^ CDS fragment and an 899 bp *ZmbHLH124* ^S‐ORG^ fragment were amplified by PCR and then cloned into the vector pCAMBIA‐2300‐Ubi‐OCS for rice transformation and the pZZ000026‐Ubi‐OCS for maize transformation, in which *ZmbHLH124* was driven by the *ZmUbiquitin1* (*GRMZM2G409726*) promoter. Transformation of maize was performed by China National Seed Group Co. Ltd., and the transformation of rice was performed by Wuhan BioRun Biotechnology Co. Ltd. For more detailed phenotypic analysis, two independent T2 transgenic positive lines were used.

To detect full‐length mRNA of *ZmbHLH124* in transgenic plants or WT seedlings, total RNA isolation and reverse transcription were performed as described above, and gene‐specific primers were used to amplify *ZmbHLH124* gene listed in Table S3 and the *ZmEF1A* gene as a control.

To detect ZmbHLH124 protein, leaves of transgenic plant or WT seedlings were collected and homogenized in liquid nitrogen. Three‐fold (v/w) of native extraction buffer (50 mm Tris‐MES pH 8.0, 0.5 m sucrose, 1 mm MgCl_2_, 10 mm EDTA, 1 mm DTT, 1 mm PMSF, protease inhibitor cocktail complete mini tablets [Roche, Basel, Switzerland] and 50 μm MG132 [Tocris Bioscience, Missouri, USA]) was added to the powder. The homogenized solutions were centrifuged at 13,523 *g* for 10 min at 4 °C. Finally, the supernatants with SDS loading buffer were boiled for 10 min, separated by SDS‐PAGE and detected by anti‐ZmbHLH124 antibody. Polyclonal antibodies of ZmbHLH124 were raised in rat.

### Transient expression in *Nicotiana benthamiana*

*Agrobacterium*‐mediated transient expression in *N. benthamiana* was carried out according to a previously described protocol (Liu *et al*., 2010). The 2329 bp‐long genomic fragment of *ZmbHLH124* ^T‐ORG^ and the 1798 bp‐long genomic fragment of *ZmbHLH124* ^S‐ORG^ were amplified separately from genomic DNA and then cloned into pCAMBIA1300‐221, in which *ZmbHLH124* variants were driven by the CaMV *35S* promoter. The 1616 bp‐long fragment for the native *ZmDREB2A* promoter (p*ZmDREB2A*) was amplified from genomic DNA, and cloned into *pENTR* using the pENTR Directional TOPO cloning kit (Invitrogen, Waltham, MA, USA). Then, p*ZmDREB2A* was fused with the luciferase reporter gene *LUC* through gateway reactions into the plant binary vector *pGWB35* (Nakagawa *et al*., 2007) to generate the reporter construct p*ZmDREB2A*:*LUC*. The effector constructs were p*35S*:*ZmbHLH124* ^T‐ORG^ and p*35S*:*ZmbHLH124* ^S‐ORG^. LUC images were captured using a low‐light cooled charge‐coupled device imaging apparatus (NightOWL II LB983 with indiGO software). For the gene expression quantification, an *Agrobacterium* strain carrying a construct encoding RFP was used as an internal control.

### Chromatin immunoprecipitation and quantitative PCR assays

To generate flag‐tag fusion protein of GFP and ZmbHLH124^T‐ORG^, the fragment containing CaMV *35S* promoter‐*flag*‐*NOS* terminator was amplified and cloned into the pBlueScript II SK(+) vector forming p*35S*:*flag* vector, then the full‐length CDS of *GFP* and *ZmbHLH124* ^T‐ORG^ were amplified and sub‐cloned into p*35S*:*flag* vector forming the flag‐tagged fusion plasmids (*flag‐GFP* and *flag‐ZmbHLH124* ^T‐ORG^). The flag‐tagged fusion plasmids were transformed into the protoplasts collected from the leaves of 10‐day‐old seedlings of inbred line B73 to perform the chromatin immunoprecipitation (ChIP) assays. The ChIP assays were conducted following a reported protocol with minor modifications (Tu *et al*., 2020). Sonicated chromatin fragments were immunoprecipitated using anti‐flag (MBL, M185‐10). The IP chromatins were detected by performing qPCR using specific primers. The formula as following was used to calculate IP%: IP% = 100 × 2 ^(−ΔCt [normalized ChIP])^, in which ΔCycle threshold ΔCt = Ct [ChIP] − (Ct [Input] − log_2_ (Input Dilution Factor)) and Input Dilution Factor = (fraction of the input chromatin saved)^−1^ (Tian *et al*., 2019). The fold enrichment was calculated against the flag control samples. The primers used in ChIP‐qPCR are listed in Table S3.

### Electrophoretic mobility shift assay

EMSAs were performed with the Chemiluminescent Nucleic Acid Detection Module (Thermo Fisher, Waltham, MA, USA) according to a previously published protocol (Wei *et al*., 2015). The coding sequence of *ZmbHLH124* ^T‐ORG^ was inserted into the *Bam*HI/*Sal*I sites of the pGEX‐6P‐1 backbone vector which contained a GST tag. The ZmbHLH124^T‐ORG^ fusion proteins were expressed at 28 °C for 3–4 h in the Rosetta *Escherichia coli* strain and purified, then GST tag was excised from the fusion protein by PreScission™ (Beyotime, Shanghai, China) Protease digestion. The single‐stranded oligonucleotide fragments of probes were synthesized and then, the double‐stranded DNAs were obtained through heating oligonucleotides at 90–95 °C in annealing solution (10 mm Tris‐HCl pH 7.5, 1 mm EDTA, 100 mm NaCl) for 5 min, then slowly cooled to room temperature. Investigation of the interaction between ZmbHLH124^T‐ORG^ protein and the corresponding probes was carried out according to the protocol provided with the LightShift™ Chemiluminescent EMSA Kit (Thermo Fisher, Waltham, MA USA). The primer sequences for constructing the GST‐ZmbHLH124^T‐ORG^ vector and the probes for the EMSA are given in Table S3.

### Stomatal density and aperture assays

For the stomatal density and aperture assays, the same regions of the second leaf from seedlings were floated on the stomatal opening buffer (20 mm KCl, 5 mm MES‐Tris and 1 mm CaCl_2_) (Wu *et al*., 2019; Xiang *et al*., 2021) for at least 1 h in the dark to ensure that most of the stomata had become fully closed. Then, after the leaf pieces maintained under light for 2 h, half of the samples were transferred to the opening buffer with 500 mm mannitol and incubated for an additional 2 h. Finally, the samples were immediately fixed by liquid nitrogen and coated with gold particles. The pictures were obtained using a scanning electron microscopy (S‐3000N; Hitachi, Tokyo, Japan), and the numbers of stomata in randomly chosen fields were counted. The width and length of the aperture for each stomatal pore were measured with ImageJ software (NIH, Bethesda, Maryland, USA).

### Accession numbers

*ZmUbiquitin1*:GRMZM2G409726, other genes and their accession numbers used in this study from MaizeGDB and Phytozome are shown in Table S3.

## Conflict of interest

The authors declare no competing financial interests.

## Author contributions

S.W., Y.W., and Q.X. designed the research. S.W., R.X., X.S., F.G., and H.W. performed the experiments and analysed the data. S.W. and C.C. carried out the transcriptomic data analyses. Y.W., R.X. and H.C. advised on the experiments. S.W., Y.W., and Q.X. wrote the manuscript.

## Supporting information


**Figure S1** Different drought phenotypes between PH4CV and F9721.
**Figure S2** Pearson correlations between different samples.
**Figure S3** Comparative analysis of chromosome regions between RIL73 and other three RILs.
**Figure S4** Comparison of two ZmbHLH124 proteins from different genetic background.
**Figure S5** Schematic diagram of NIL development.
**Figure S6** Expression level analyses of maize transgenic lines.
**Figure S7** Effects of ZmbHLH124 overexpression on stomatal movement.
**Figure S8** Phenotypes of ZmbHLH124 transgenic rice plants upon drought treatment.

**Table S1** Genotypes of four RILs.

**Table S2** Annotations of differentially expressed transcription factors in RIL73 specific regions.

**Table S3** Primers used in this study.

## Data Availability

The raw sequence data reported in this paper have been deposited in the National Center for Biotechnology Information Sequence Read Archive (accession no. PRJNA662679).
