# Supplementary material for: ZmbHLH124 identified in maize recombinant inbred lines contributes to drought tolerance in crops
Source: Plant Biotechnol J. 2021 Jul 6;19(10):2069–81. doi: 10.1111/pbi.13637 (PMC8486247; doi:10.1111/pbi.13637)
Supplement: Supplementary file 1 — Figure S1 Different drought phenotypes between PH4CV and F9721. Figure S2 Pearson correlations between different samples. Figure S3 Comparative analysis of chromosome regions between RIL73 and other three RILs. Figure S4 Comparison of two ZmbHLH124 proteins from different genetic background. Figure S5 Schematic diagram of NIL development. Figure S6 Expression level analyses of maize transgenic lines. Figure S7 Effects of ZmbHLH124 overexpression on stomatal movement. Figure S8 Phenotypes of ZmbHLH124 transgenic rice plants upon drought treatment. [file PBI-19-2069-s001.pdf]

Figure S1

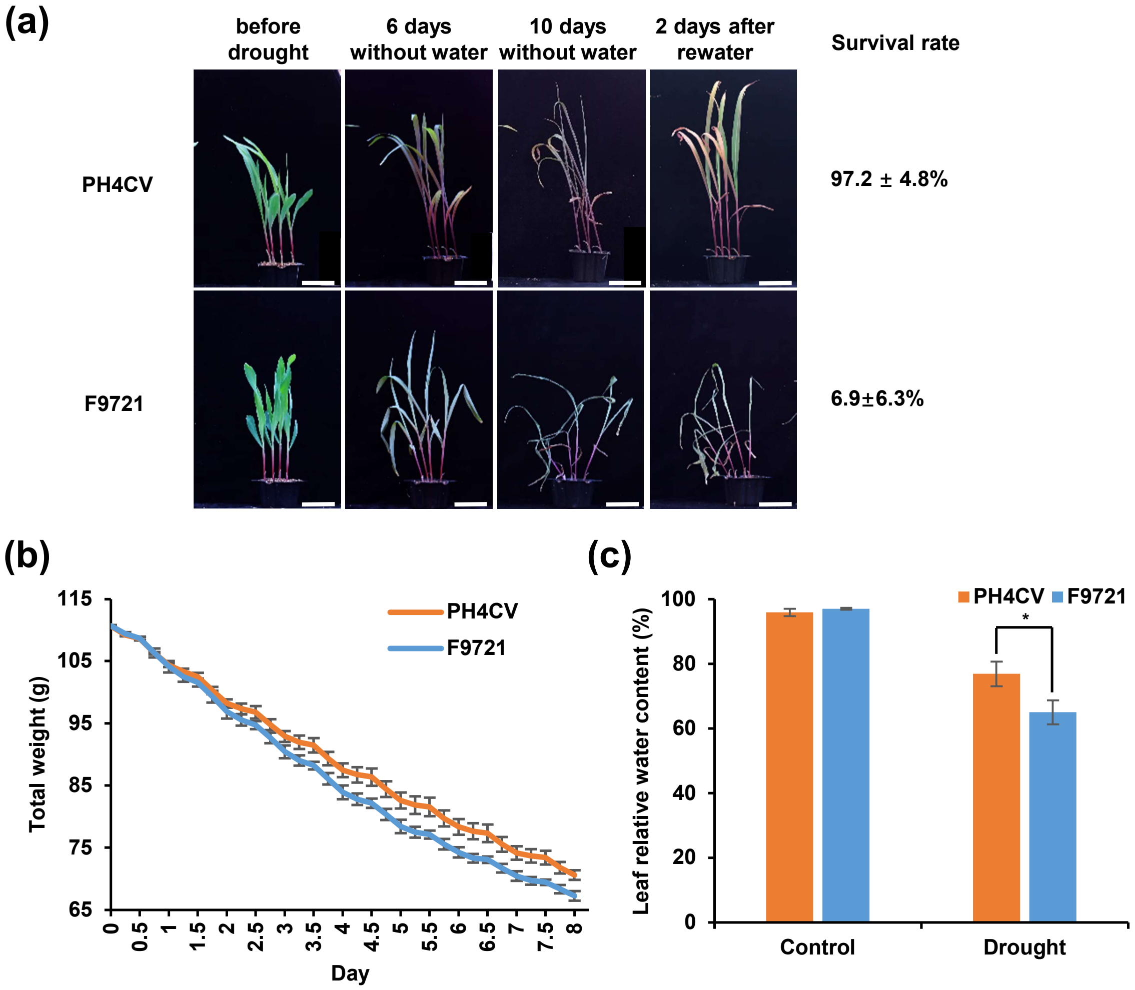

Figure S1 Different drought phenotypes between PH4CV and F9721. (a) The phenotype changes of PH4CV and F9721 during drought stress. Bar = 5 cm. (b) Water loss rates of seedlings during the drought treatment. Y-axis represents the total weight of pots, soil and seedlings. (c) Leaf relative water content. The leaves were collected when the soil moisture content is about 12%. Error bars, s.d., calculated from the results of at least three independent experiments; statistical significance was determined by a two-tailed *t*-test: $*P < 0.05$, $**P < 0.01$.

Figure S2

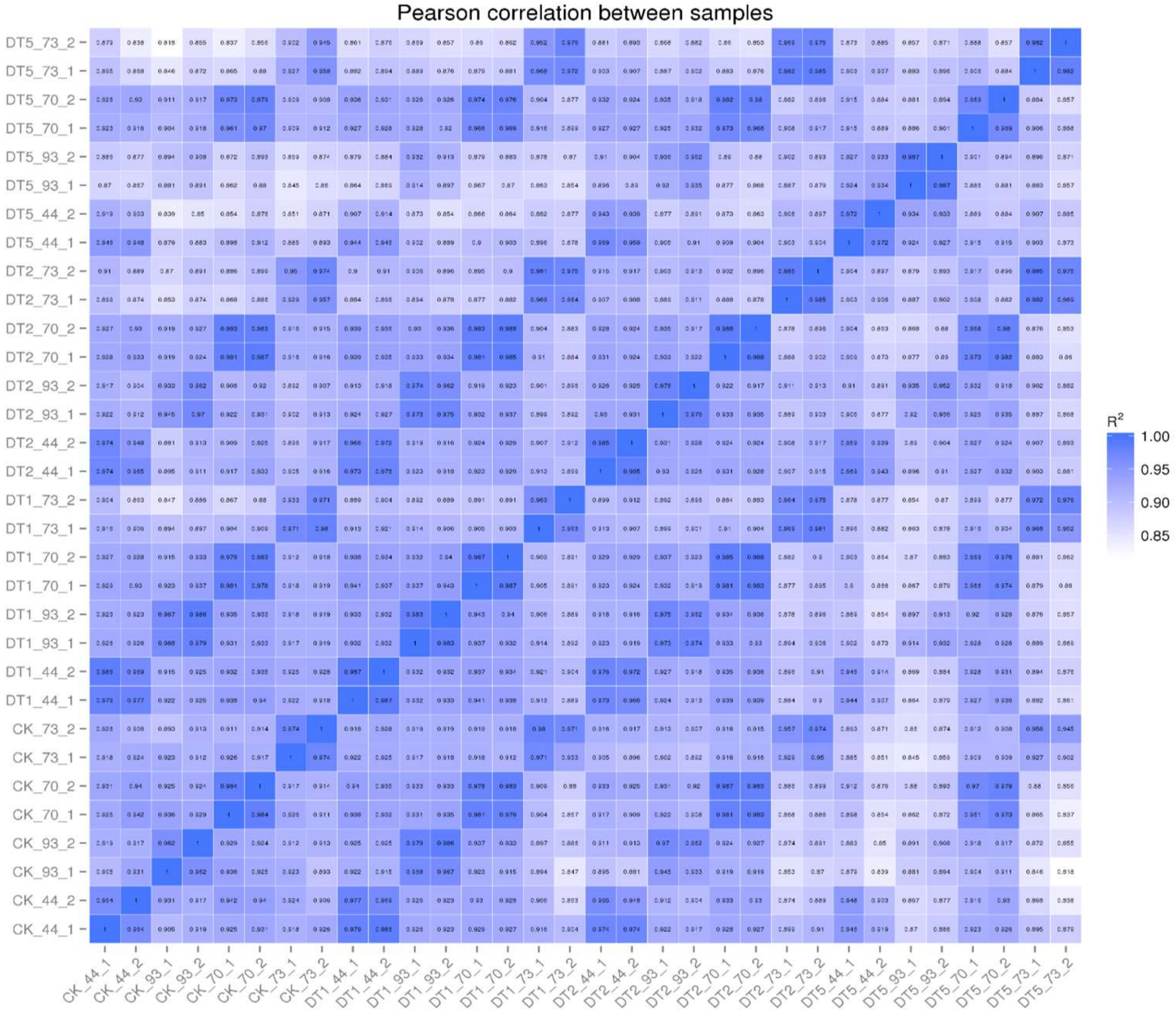

Figure S2 The Pearson correlations between different samples. CK, DT1, DT2, and DT5 refer to drought treatment for 0, 1, 2 and 5 days respectively.

Figure S3

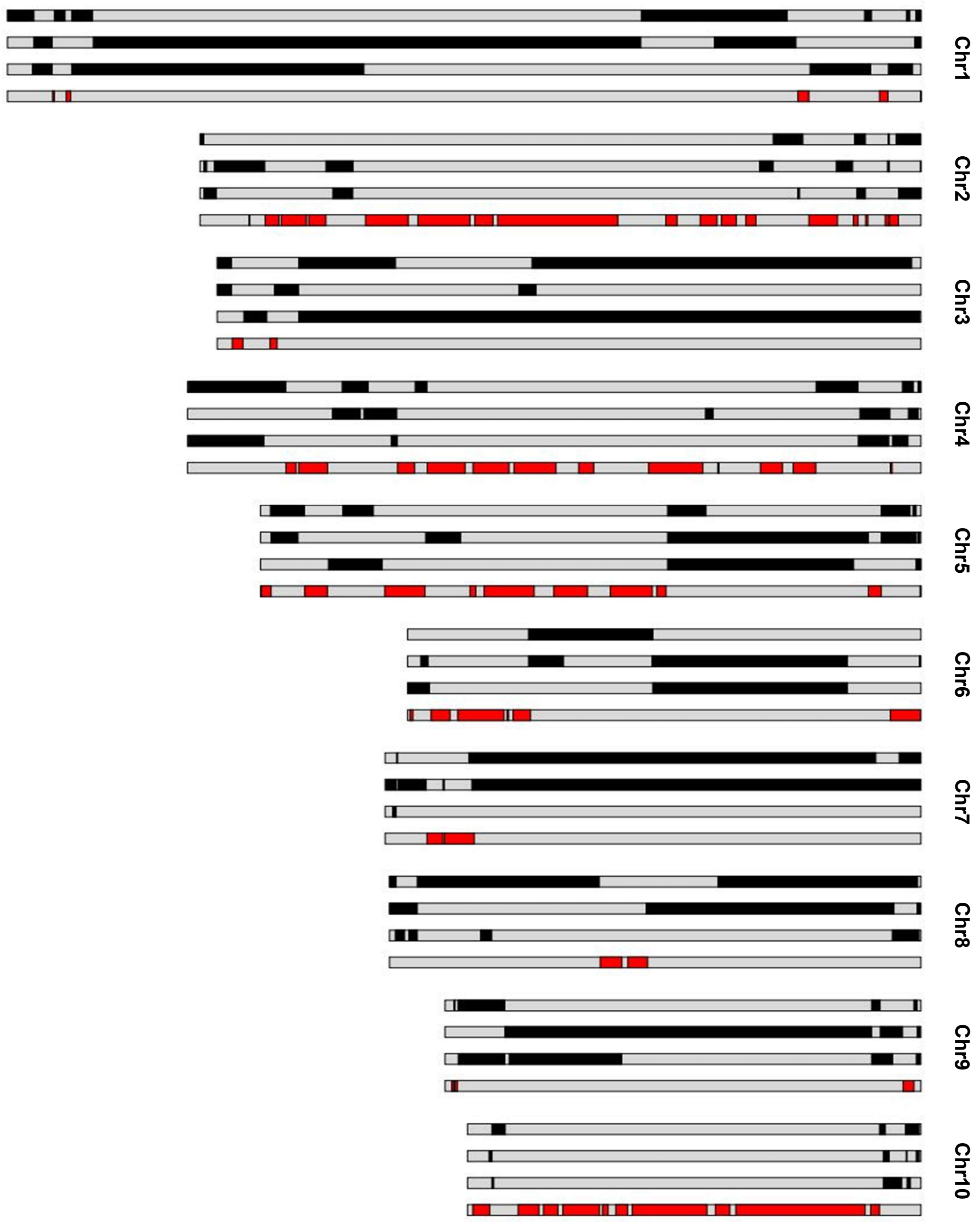

Figure S3 Comparative analysis of chromosome regions between RIL73 and other three RILs. Under one chromosome, the four columns from top to bottom represent the chromosome from RIL44, RIL93, RIL70 and RIL73 respectively. Black boxes represent common regions with similar genetic backgrounds between RIL73 and RIL44, RIL93 or RIL70, red boxes represent RIL73 specific regions different from other three RILs.

Figure S4

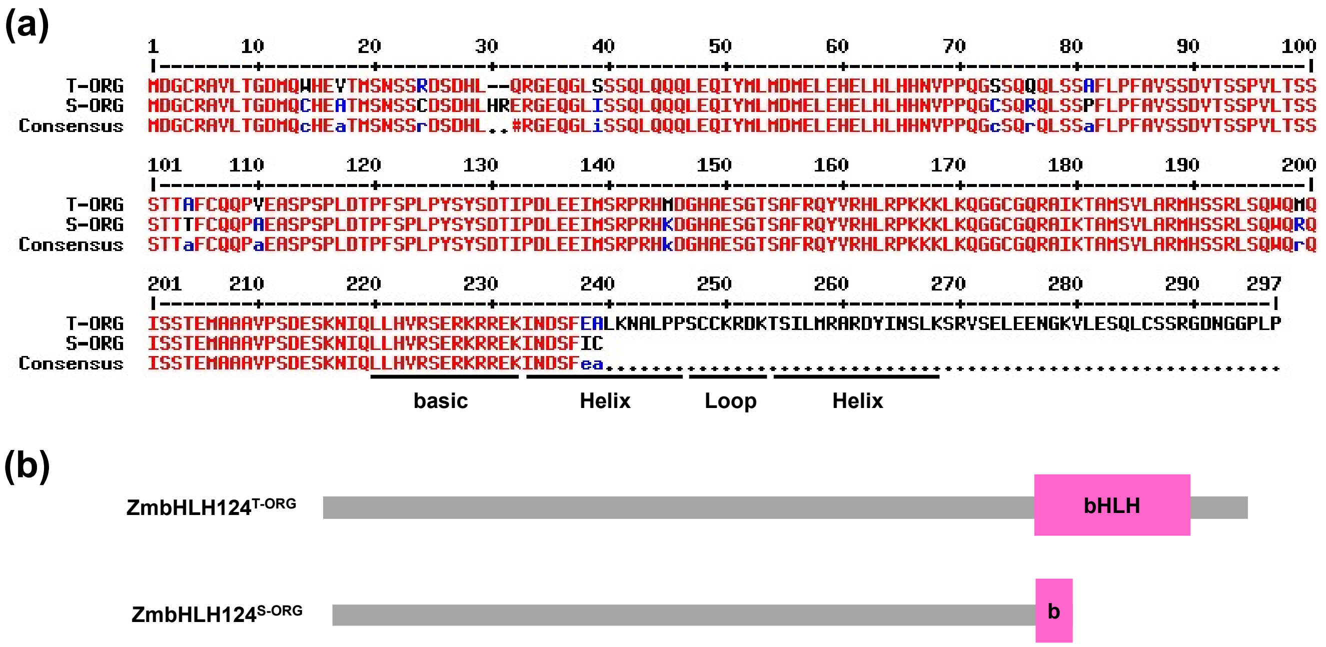

Figure S4 Comparison of two ZmbHLH124 proteins from different genetic background. (a) Alignment of ZmbHLH124 amino acid sequences from two parental lines. Red letters indicate consensus amino acids between two proteins, blue letters indicate different amino acids between two proteins. T-ORG here indicates ZmbHLH124$^{T\text{-}ORG}$ protein, and S-ORG here indicates ZmbHLH124$^{S\text{-}ORG}$ protein. (b) Schematic diagram of ZmbHLH124 protein structures. Gray boxes represent protein primary structure, colored boxes represent domains within proteins.

Figure S5

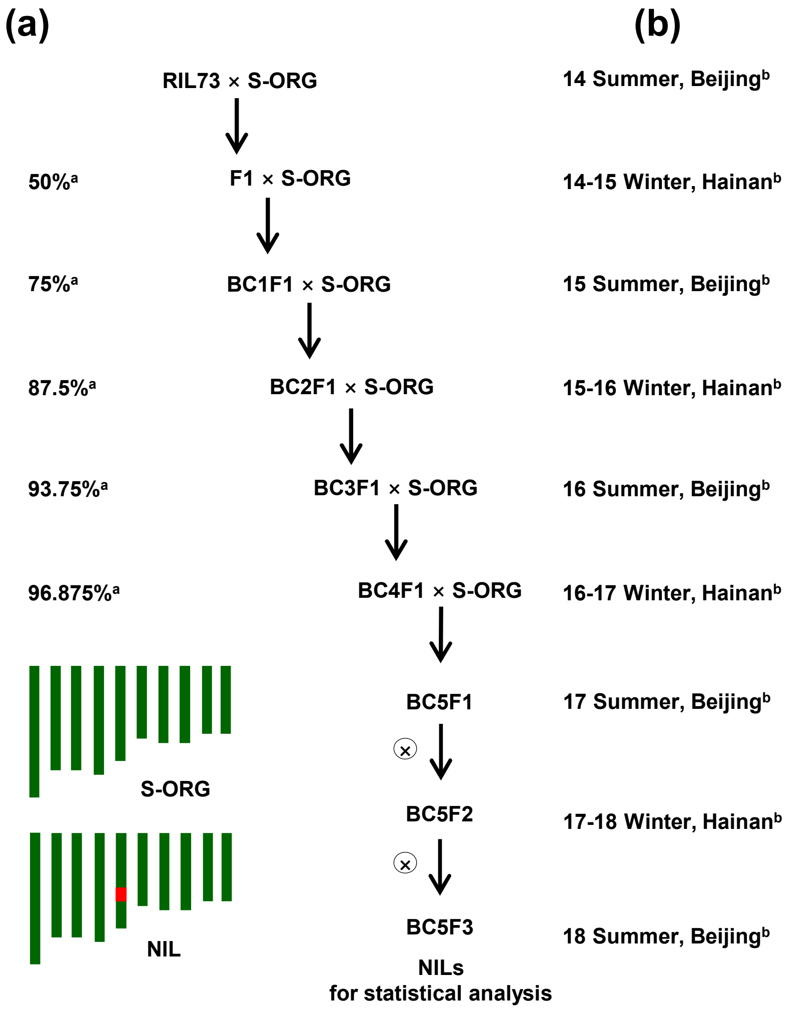

Figure S5 Schematic diagram of NIL development. Foreground selection using the flanking markers and background selection using 133 polymorphic markers. a: Ratio of S-ORG (F9721) background in every backcross generation; b: Time to backcross or self-pollination.

Figure S6

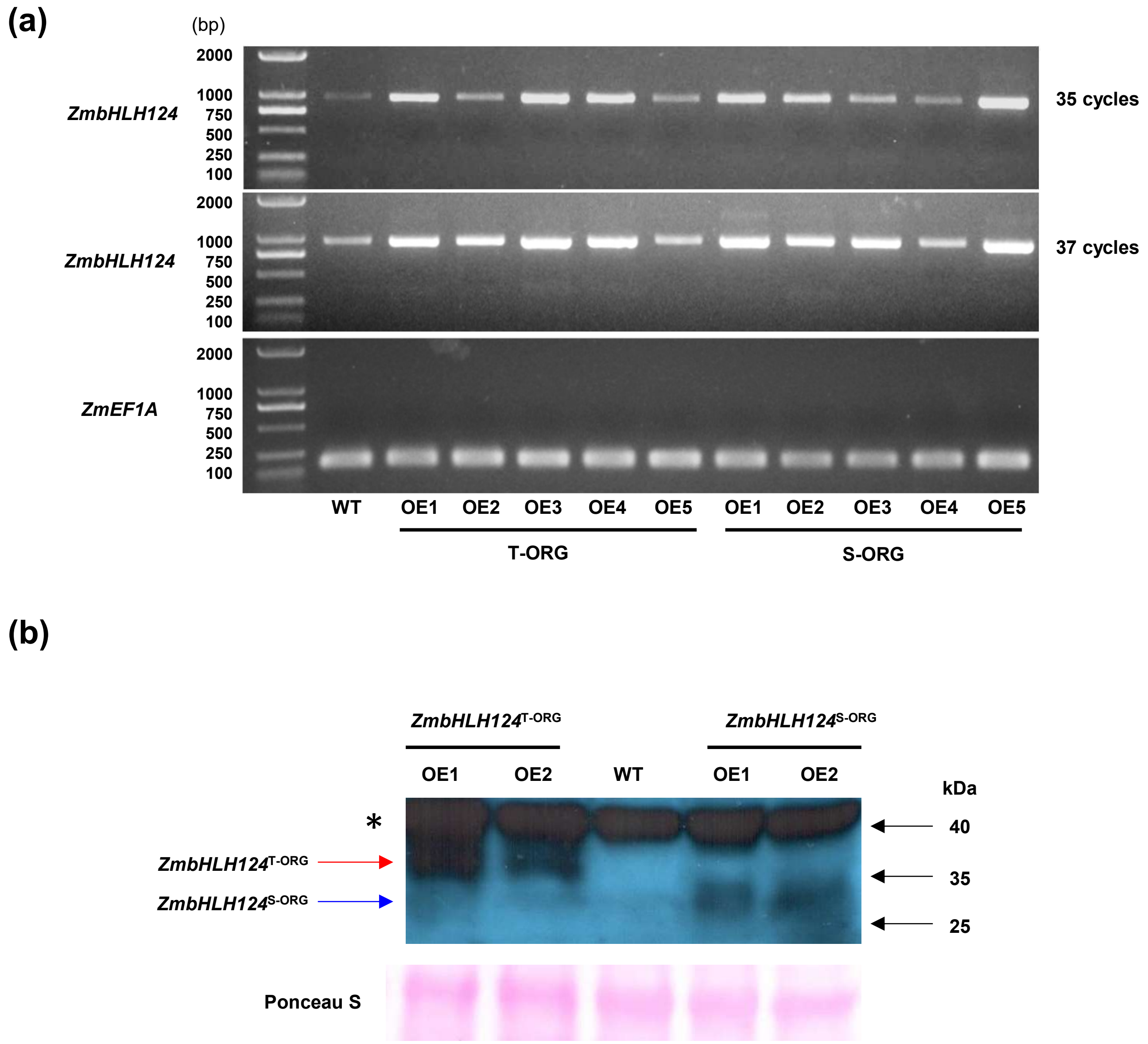

Figure S6 Expression level assays of transgenic lines. (a) Semi-qPCR assay of *ZmbHLH124* RNA level of transgenic lines and *ZmEF1A* gene used as a control. (b) Western blot assay of ZmbHLH124 protein level of transgenic lines and Ponceau S used as the loading control. * indicates the unspecific band.

Figure S7

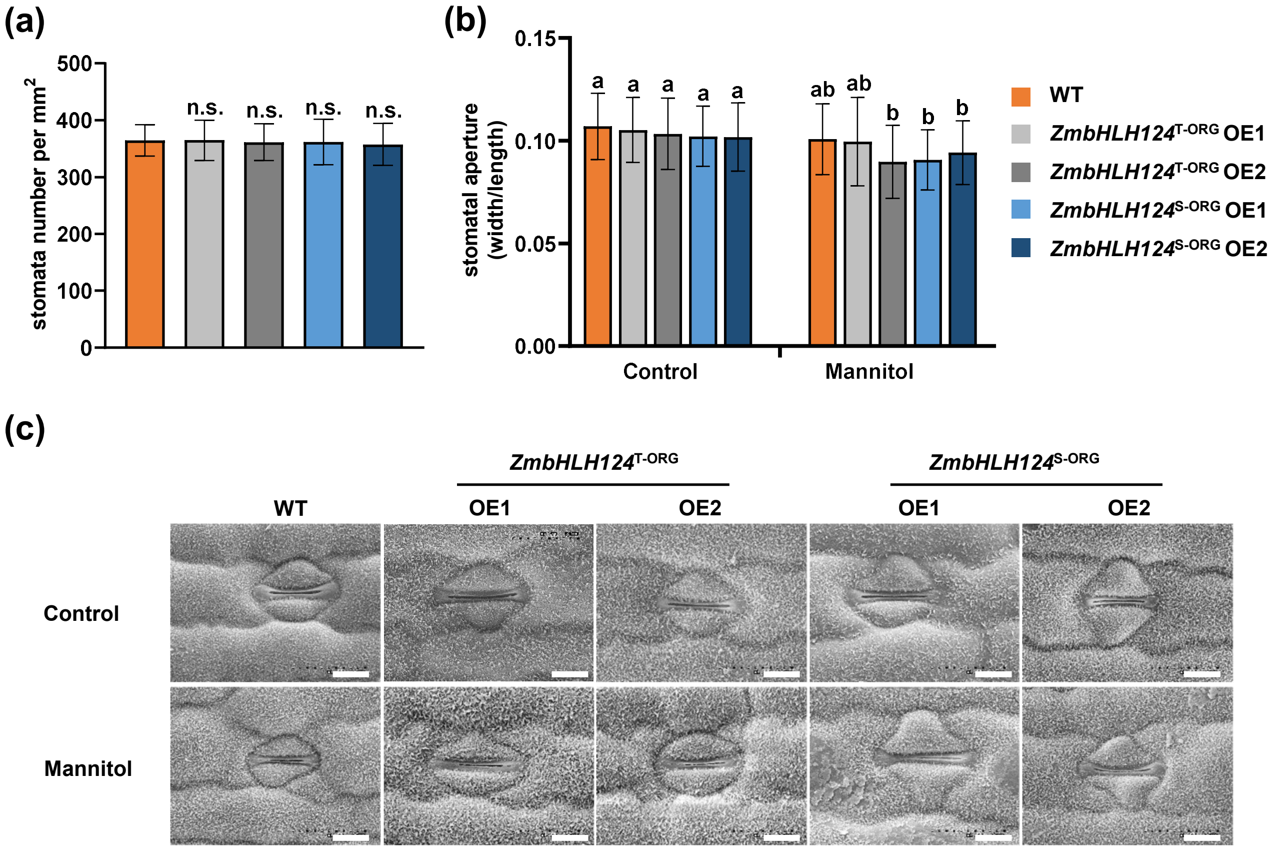

Figure S7 Effects of *ZmbHLH124* overexpression on stomata. (a) Stomatal density. Ten leaves were used and around 4 views were visualized, totally at least 40 pictures were taken. Error bars, s.d.. Statistical significance compared with WT was determined by a two-sided *t*-test. (b) Stomatal aperture. Ten leaves were used and around 6 stomata were visualized, totally at least 60 stomata were calculated. Statistical significance was determined by one-way ANOVA multiple range tests. (c) Images of the abaxial epidermis of the second leaf of plants at the three-leaf stage for the wild-type (WT) and *ZmbHLH124* overexpression lines. Bar = 15 μm. Control: opening buffer; Mannitol: opening buffer with 500 mM mannitol.

Figure S8

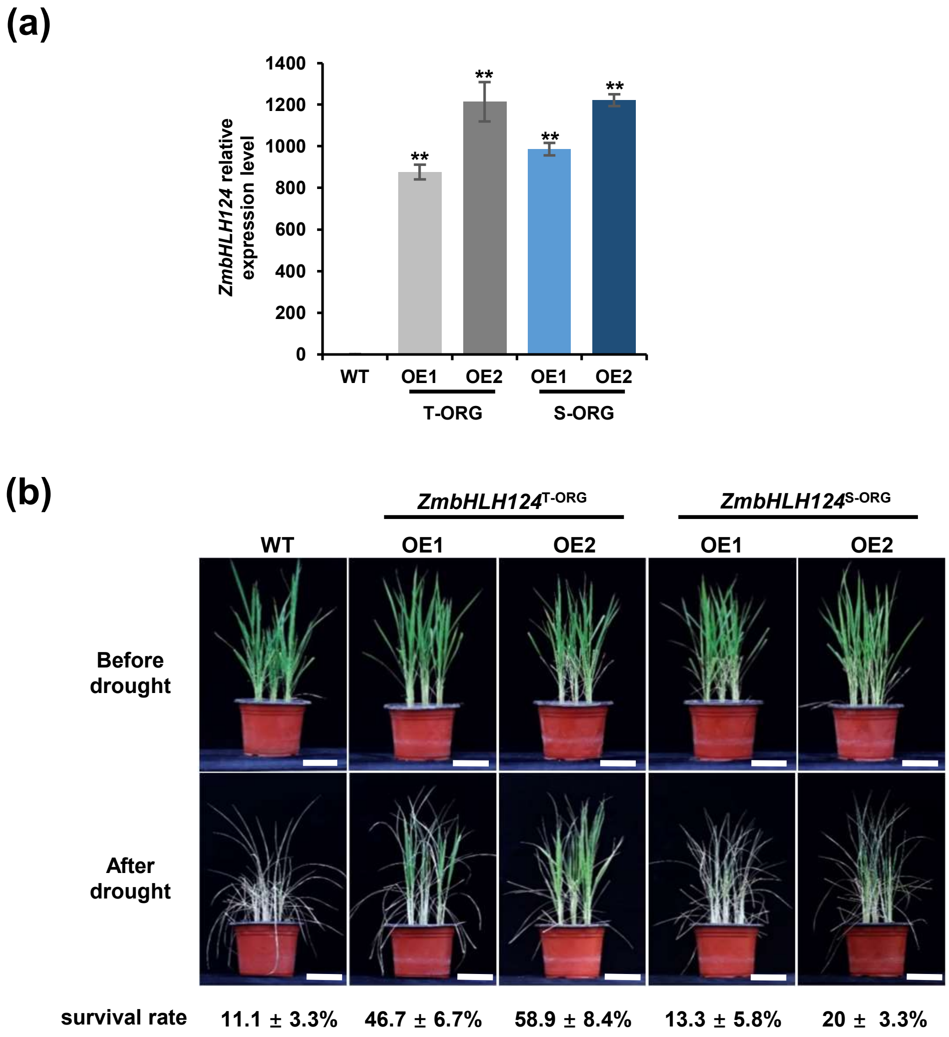

Figure S8 Phenotypes of *ZmbHLH124* transgenic rice upon drought treatment. (a) Relative expression levels of *ZmbHLH124* detected by qRT-PCR in rice grown under normal conditions. The data were based on three independent biological replicates. Error bars, s.d.. Statistical significance compared with WT was determined by a two-sided *t*-test: $^*P < 0.05$, $^{**}P < 0.01$. (b) Drought treatment of transgenic rice as compared to WT in different time points. Survival rates were calculated from the results of at least three independent experiments, $n \geq 30$. Bar = 8 cm.
