# Supplementary material for: ZmbHLH124 identified in maize recombinant inbred lines contributes to drought tolerance in crops
Source: Plant Biotechnol J. 2021 Jul 6;19(10):2069–81. doi: 10.1111/pbi.13637 (PMC8486247; doi:10.1111/pbi.13637)
Supplement: Supplementary file 4 — Table S3 Primers used in this study. [file PBI-19-2069-s002.docx]

**Primers for vector construction**

| **Vector name** | **Primer name** | **Primer sequences (5'-3')** |
| --- | --- | --- |
| p*Ubi*:*ZmbHLH124*^PH4CV^ | *ZmbHLH124*-S-Psc | cgactctagaggatcaattcgagctccATGGATGGTTGCAGAGCTG |
|  | *ZmbHLH124*-A-Psc | gcctgcaggtcgactctagaggatccTTACGGCAATGGTCCTCC |
| p*Ubi*:*ZmbHLH124*^F9721^ | *ZmbHLH124*-S-Fsc | cgactctagaggatcaattcgagctcATGTCACGAGGCGACGATG |
|  | *ZmbHLH124*-A-Fsc | gcctgcaggtcgactctagaggatccTTACGGCAATGGTCCTGC |
| p*35S*:*ZmbHLH124*^PH4CV^ | *ZmbHLH124*-Fsc | ttggagagaacacgggggactctagaGATCTCGCCATGGATGGTTG |
|  | P*-ZmbHLH124*-Rsc | gaacgatcggggaaattcgagctcTTAGGGTGTCTCTATCTCTGAC |
| p*35S*:*ZmbHLH124*^F9721^ | F*-ZmbHLH124*-Rsc | gaacgatcggggaaattcgagctcTAAGTTGTACCAAACATGGC |
| p*ZmDREB2A*:*LUC* | *ZmDREB2A-*Pro-F | caccTCACTGATATAGGCACACCGTCACCAC |
|  | *ZmDREB2A*-Pro-Rtopo | GGCCGCTCCAAAGATGGCCACT |

**Primers for qRT-PCR**

| **Gene** | **Primer names** | **Primer sequences (5'-3')** |
| --- | --- | --- |
| GRMZM2G153541 | *EF1A* qF | TGGGCCTACTGGTCTTACTACTGA |
|  | *EF1A* qR | ACATACCCACGCTTCAGATCCT |
| GRMZM2G041761 | 041761-qF | GTATCAGTTCATTCGTGTTC |
|  | 041761-qR | ATCGTGGTAAGAGTAGAGT |
| GRMZM2G042895 | 042895-qF | GGTGCTTTGTTTGCTATA |
|  | 042895-qR | TTCGGTCCATTACATCAA |
| GRMZM2G081782 | 081782-qF | ATCACCGAAGTTATACATCA |
|  | 081782-qR | AACACCTTTAGCACAGAG |
| GRMZM2G091044 | 091044-qF | CACCACTCCATCCAGATA |
|  | 091044-qR | GTCCAGCACCTTCTGTAT |
| GRMZM2G125775 | 125775-qF | TAAGGCAGATGTGAAGAC |
|  | 125775-qR | TAGTGTGGCAATATGAATTAC |
| GRMZM2G132550 | *ZmbHLH124* qF | GCGACTCTGATCATCTTC |
|  | *ZmbHLH124* qR | TCTAGCTCCATGTCCATC |
| GRMZM2G155217 | 155217-qF | TAGGTAGAAGAAGGATGAGA |
|  | 155217-qR | TGTGGTAACGAGGTAGTA |
| GRMZM5G801627 | 801627-qF | CTCCGACCTCATCAACTG |
|  | 801627-qR | ACCATCCAAGACTATTCACAA |
| GRMZM2G006745 | *ZmDREB2A* qF | TTGAAGCCTCTGACATCTC |
|  | *ZmDREB2A* qR | AACCATCACCACCATCTG |
| GRMZM2G172230 | *ZmERD1* qF | CACGATACTGATGGACAT |
|  | *ZmERD1* qR | CCTAATCTACTACACTGGAA |
| GRMZM5G872256 | *ZmGolS3* qF | CGGAACATAAGAAGAATCTCT |
|  | *ZmGolS3* qR | GCTCCACACTTACACAAG |
| GRMZM2G479760 | *ZmABF2* qF | TTGTGCCTCCACTGCAATTCGG |
|  | *ZmABF2* qR | TTACAAGTCGTCTCCCTCCATCTTCC |
| GRMZM2G098750 | *ZmRAB18* qF | GTGGTGGGTTGAGGGGAAGGAAGC |
|  | *ZmRAB18* qR | ACGCCATCGCCGTTGAGCCT |
| LOC_Os03g50885 | *OsACTIN* qF | TGGTCGTACCACAGGTATTGTGTT |
|  | *OsACTIN* qR | AAGGTCGAGACGAAGGATAGCAT |

**Primers for EMSA**

| **Primer name** | **Primer sequences (5'-3')** |
| --- | --- |
| p*ZmDREB2A-*F | TCGCTCTCACTTCAATACACCACGTCcaCGtgAGCCTGTTCTGTTCCAC  TGACACACG |
| p*ZmDREB2A-*R | CGTGTGTCAGTGGAACAGAACAGGCTcaCGtgGACGTGGTGTATTGAA  GTGAGAGCGA |
| p*ZmDREB2A-*F-mu1,2 | TCGCTCTCACTTCAATACACCACGTCttCGccAGCCTGTTCTGTTCCAC  TGACACACG |
| p*ZmDREB2A-*R-mu1,2 | CGTGTGTCAGTGGAACAGAACAGGCTggCGaaGACGTGGTGTATTGA  AGTGAGAGCGA |
| p*ZmDREB2A-*F-mu2,3 | TCGCTCTCACTTCAATACACggaaaCcaCGtgAGCCTGTTCTGTTCCA  CTGACACACG |
| p*ZmDREB2A-*R-mu2,3 | CGTGTGTCAGTGGAACAGAACAGGCTcaCGtgGtttccGTGTATTGAA  GTGAGAGCGA |
| p*ZmDREB2A-*F-mu1,2,3 | TCGCTCTCACTTCAATACACggaaaCttgGccAGCCTGTTCTGTTCCACTGA  CACACG |
| p*ZmDREB2A-*R-mu1,2,3 | CGTGTGTCAGTGGAACAGAACAGGCTggCcaaGtttccGTGTATTGAA  GTGAGAGCGA |

**Primers for ChIP-qPCR**

| **Primer name** | **Primer sequences (5'-3')** |
| --- | --- |
| P1*-*F | CCTTACGTCAGCTCGCTCTCA |
| P1*-*R | CGTGGGTTCCGTGTGTCAGT |
| P2*-*F | GTCCTCTAAATCACATCCTCTATACTCAA |
| P2*-*R | GGACGCTGCTGGATTCTCTA |
| P3*-*F | GAATAACTTTCATGCAACGG |
| P3*-*R | TGGAGAGGAAGGAGATATAG |
| P4*-*F | ATGTCGCTGTTCACCAA |
| P4*-*R | GATGAAGCCCAAATATGAGT |

**Other primers**

| **Applicatin** | **Primer name** | **Primer sequences (5'-3')** |
| --- | --- | --- |
| NIL construction | *bHLH124*-idF | GAGGAGACAACGCAGGACCATT |
|  | *bHLH124*-idR | GAAGCATCAACAGAACATCAGACGAA |
| DNA quantification reference | *NbACT*-F | CATCAGGAAGGACTTGTACGG |
|  | *NbACT*-R | GATGGACCTGACTCGTCATAC |
| Transactivation assay internal control | *RFP*-qF | CAACGAGGACTACACCAT |
|  | *RFP*-qR | GGCAACAGGATTCAATCTTAA |
| Promoter DNA detection | p*ZmDREB2A*-dF | TTAGAGGACGCTACTAGAG |
|  | p*ZmDREB2A*-dR | TGTGTTCTTGTTGGAGTC |
| Protein expression | ZmbHLH124-*Bam*HI-F | CGggatccATGGATGGTTGCAGAGCTG |
|  | ZmbHLH124-*Sal*I-R | GCgtcgacCGGCAATGGTCCTCCGTT |
| Semi qPCR | *ZmbHLH124*-cdsF | ATGGATGGTTGCAGAGCTGTC |
|  | *ZmbHLH124*-cdsR | TTACGGCAATGGTCCTCCGTT |
| Semi qPCR | ZmEF1A-cdsF | CTTGGTGTGAAGCAGATG |
|  | ZmEF1A-cdsR | CAGAGATTGGAACGAAGTG |
